# Patterns of health care utilization related to initiation of amitriptyline, duloxetine, gabapentin, or pregabalin in fibromyalgia

**DOI:** 10.1186/s13075-015-0530-8

**Published:** 2015-01-28

**Authors:** Seoyoung C Kim, Joan E Landon, Yvonne C Lee

**Affiliations:** Division of Pharmacoepidemiology and Pharmacoeconomics, Brigham and Women’s Hospital and Harvard Medical School, 1620 Tremont St, Suite 3030, Boston, MA 02120 USA; Division of Rheumatology, Immunology and Allergy, Brigham and Women’s Hospital and Harvard Medical School, 1620 Tremont St, Suite 3030, Boston, MA 02120 USA

## Abstract

**Introduction:**

Several pharmacologic treatments are available for fibromyalgia, but little is known about the comparative effectiveness of these treatments on health care utilization.

**Methods:**

Using US commercial insurance claims data (covering 2007 to 2009), we conducted a cohort study to examine the comparative effectiveness of amitriptyline, duloxetine, gabapentin, and pregabalin on health care utilization in patients with fibromyalgia. We measured patients’ medication adherence using the proportion of days covered (PDC) and estimated multivariable rate ratios (RRs) for outpatient visits, prescriptions, hospitalization, and emergency department (ED) visits in propensity score (PS)–matched cohorts.

**Results:**

Cohorts of 8,269 amitriptyline, 9,941 duloxetine, and 18,613 gabapentin initiators were compared with their PS-matched pregabalin initiators. During the baseline 180-day period, patients had, on average, seven to nine physician visits, including six to eight specialist visits, and received eight prescription drugs. The mean PDC up to 180 days varied from 38.6% to 67.7%. The number of outpatient visits, prescriptions, and hospitalizations decreased slightly after initiating one of the study drugs, but the number of ED visits increased after treatment initiation. Compared to pregabalin, duloxetine was associated with decreased outpatient visits (RR, 0.94; 95% confidence interval (CI), 0.88 to 1.00), prescriptions (RR, 0.94; 95% CI, 0.90 to 0.98), hospitalizations (RR, 0.75; 95% CI, 0.68 to 0.83), and ED visits (RR, 0.85; 95% CI, 0.79 to 0.91). Little difference in health care utilization rates was noted among amitriptyline and gabapentin initiators compared to those who were started on pregabalin.

**Conclusions:**

Fibromyalgia patients had high health care utilization before and after initiation of amitriptyline, duloxetine, gabapentin, or pregabalin. Medication adherence was suboptimal. Overall, fibromyalgia treatment had little impact on reducing health care utilization, but duloxetine initiators had less health care utilization than those started on pregabalin.

**Electronic supplementary material:**

The online version of this article (doi:10.1186/s13075-015-0530-8) contains supplementary material, which is available to authorized users.

## Introduction

Fibromyalgia is a common, highly disabling syndrome characterized by chronic widespread pain, fatigue, and problems with sleep, memory, and mood [1-3]. Treatment of fibromyalgia is challenging and often requires both pharmacologic and nonpharmacologic treatments [4,5]. For pharmacologic treatment, tricyclic antidepressants (TCAs) such as amitriptyline, selective serotonin reuptake inhibitors (SSRIs), serotonin and noradrenaline reuptake inhibitors (SNRIs) such as duloxetine and milnacipran, and monoamine oxidase inhibitors are used to reduce pain, depression, fatigue, and health-related quality of life [6,7]. Gabapentin and pregabalin are also commonly prescribed to ameliorate pain and sleep problems associated with fibromyalgia [6,7].

Patients with fibromyalgia are known to be heavy users of health care systems, including outpatient visits, hospitalizations, and prescription drug use [8-10]. Despite the number of available drugs for fibromyalgia, few studies have directly compared the effects of different pharmacologic agents for fibromyalgia on health care utilization. In studies comparing pre- and posttreatment, pregabalin initiators had increased inpatient and outpatient visits after initiating pregabalin, whereas duloxetine initiators had decreased inpatient and outpatient visits after initiating duloxetine [11-13]. In another study, researchers reported increases in total health care costs after initiating treatment with pregabalin or duloxetine for fibromyalgia [10]. In a study comparing pregabalin to TCAs, pregabalin initiators had increased total health care costs from pretreatment to follow-up [14]. Although all of these studies were performed using US claims data, the effects of fibromyalgia treatment on health care utilization may vary across different health care systems, particularly across different countries.

The objective of this study was to examine the comparative effectiveness of four commonly used drugs for fibromyalgia—amitriptyline, duloxetine, gabapentin, and pregabalin—on the rate of health care utilization in a US population-based cohort of commercially insured patients with fibromyalgia. Patients’ adherence to these drug regimens (that is, continuation or discontinuation) was also assessed. We hypothesized that the effect of amitriptyline, duloxetine, or gabapentin on health care utilization would differ from the effect of pregabalin.

## Methods

### Data source

We conducted a cohort study using claims data obtained from UnitedHealthcare, a US commercial health insurance plan, which insures primarily working adults and their family members, for the period from 1 January 2007 to 31 December 2009. The details of the data source are presented elsewhere [15]. The study protocol was approved by the Institutional Review Board of the Brigham and Women’s Hospital. Patients’ informed consent was not required by the Institutional Review Board, as the dataset was deidentified to protect subject confidentiality.

### Study cohort

Among patients ages 18 years and older who had at least one visit coded with the International Classification of Diseases, Ninth Revision, Clinical Modification (ICD 9-CM), code 729.1x, for fibromyalgia, new users of a study drug—amitriptyline, duloxetine, gabapentin, or pregabalin—were identified. Milnacipran users were initially considered, but they were subsequently excluded because of the small number of patients taking milnacipran (less than 0.5% of all subjects). To be included in the study cohort, we required patients to have at least 180 days of continuous health plan eligibility before receiving the first prescription of a study drug. Patients were required to be naïve to all four drugs during the 180-day baseline period. We excluded patients who were prescribed more than one study drug at the same index date.

Follow-up time started on the index date, defined as the dispensing date of the first study drug, and ended at the first of any of the following censoring events: discontinuation or switching of study drugs, loss of health plan eligibility, end of study database, or death.

### Health care utilization

Changes in health care utilization factors (for example, the number of outpatient visits to any physicians, primary care physicians and any specialists; number of prescription drugs; acute care hospitalizations; emergency department (ED) visits; physical therapy) were assessed during follow-up.

### Covariates

Variables potentially related to fibromyalgia symptoms, initiation of the study drugs, or health care utilization were assessed using data from the 180-day baseline period. These variables included age, sex, fibromyalgia-related comorbidities (back pain, chronic headache, depression, anxiety, chronic pain, fatigue, sleep disorder, and abdominal pain condition), other comorbidities (hypertension, diabetes, stroke, cardiovascular disease, malignancy, and inflammatory arthritis), use of other medications (opioids, benzodiazepines, anticonvulsants excluding gabapentin and pregabalin, TCAs excluding amitriptyline, SSRIs, SNRIs excluding duloxetine, other antidepressants, muscle relaxants, nonbenzodiazepine sleep disorder drugs, oral glucocorticoids, nonsteroidal anti-inflammatory drugs (NSAIDs), topical analgesics, migraine drugs, and gastrointestinal protective drugs), and health care utilization factors (number of outpatient visits to any physicians, primary care physicians, and specialists; number of prescription drugs; physical therapy; ED visits; and acute hospitalizations) [15,16]. Additional files 1 and 2 contain the lists of diagnoses and procedure codes and medications. To quantify patients’ comorbidities, we also calculated the Deyo-adapted Charlson Comorbidity Index based on the ICD-9-CM [17,18].

### Statistical analysis

We compared the baseline characteristics among the initiators of each study drug. To control for potential confounding by indication, we used propensity score (PS) estimation [19]. In multivariable logistic regression models using pregabalin initiators as the common reference, we estimated a PS. Pregabalin was chosen as the common reference because (1) we wanted to directly compare one treatment approved by the US Food and Drug Administration (FDA) to another FDA-approved treatment (duloxetine) and other non-FDA-approved treatments (amitriptyline and gabapentin), and (2) the number of pregabalin initiators was greater than that of duloxetine initiators. All the aforementioned baseline covariates except health care utilization factors were included in the logistic models. Each pair—amitriptyline versus pregabalin, duloxetine versus pregabalin, and gabapentin versus pregabalin—was then matched on the PS using the greedy matching algorithm [20]. To measure patients’ adherence to their fibromyalgia treatments, we calculated the proportion of days covered (PDC) as the total number of days’ supply provided by prescriptions for a given study drug up to 180 days from the index date divided by 180 days.

Adjusting for baseline health care utilization factors, we used multivariable Cox models to estimate rate ratios (RR) for ED visits, acute care hospitalizations, and physical therapy during the follow-up period in all three PS-matched pairs [21]. Multivariable Poisson regression, adjusted for baseline health care utilization factors, was used to estimate RR for outpatient visits to any physician, primary care physicians, and any specialists, as well as the number of prescription drugs, during the follow-up period in all three PS-matched pairs. To deal with the potential overdispersion in Poisson models, the standard errors were adjusted with the scale option in SAS 9.3 software (SAS Institute, Cary, NC, USA). To assess a long-term effect of treatment, a subgroup analysis using multivariable Poisson and Cox regression models was performed in patients with treatment duration of at least 180 days. All analyses were done using SAS 9.3 software.

## Results

### Study cohort

Among 116,183 patients who had at least one diagnosis of fibromyalgia between 2007 and 2009, we identified 13,404 amitriptyline, 18,420 duloxetine, 23,268 gabapentin, and 19,286 pregabalin initiators [15]. Of those, 8,269 amitriptyline, 9,941 duloxetine, and 18,613 gabapentin initiators were matched to pregabalin initiators on their PS with a ratio of 1:1. Thus, different subsets of pregabalin users were compared for each pairwise analysis.

### Baseline characteristics

Demographic characteristics, comorbidities, and medications at baseline were well balanced and statistically not different between the PS-matched cohorts (Table 1). The mean age ranged from 47.7 to 50.0 years, depending on the treatment pairs. The majority of patients were women (77% to 82%). The median starting daily doses were 25 mg for amitriptyline, 60 mg for duloxetine, 300 mg for gabapentin, and 75 mg for pregabalin. Back pain was the most common comorbidity (52% to 63%), followed by hypertension (30% to 36%). During the 180-day baseline, more than one-half of patients received at least one prescription for opioids and one-third used benzodiazepines. Use of SSRIs, non-SSRIs/SNRIs antidepressants, muscle relaxants, oral steroids, and NSAIDs was also common.

**Table 1:** **Baseline characteristics of the propensity score–matched cohorts in the 180 days prior to initiation of amitriptyline, duloxetine, gabapentin, or pregabalin** ^**a**^

		**Amitriptyline**	**Pregabalin**	**Duloxetine**	**Pregabalin**	**Gabapentin**	**Pregabalin**
Number of patients		8,269	8,269	9,941	9,941	18,613	18,613
Demographics							
	Age (yr)	47.7 ± 12.4	47.7 ± 11.7	48.3 ± 11.6	48.2 ± 11.4	50.0 ± 12.6	50.0 ± 11.8
	Female	79.6	79.8	82.0	82.0	77.3	77.6
Comorbidities							
	Back pain	52.4	52.5	55.3	55.7	63.2	63.3
	Headache	26.0	26.3	22.2	22.6	21.6	21.7
	Depression	10.6	10.5	16.3	16.7	12.0	12.1
	Anxiety	12.6	12.7	15.9	16.6	13.0	13.0
	Abdominal pain	4.4	4.3	4.2	4.0	3.5	3.6
	Neuropathic pain	25.4*	33.4*	30.4*	36.1*	41.6*	40.5*
	Sleep disorder	16.7	16.2	18.3	18.4	16.5	16.5
	Diabetes	11.5	11.3	12.7	12.8	15.9	15.8
	Hypertension	29.8	30.0	31.6	31.2	35.7	35.7
	Cardiovascular disease	2.0	2.0	2.0	2.1	3.0	2.9
	Stroke	3.2	3.1	3.2	3.0	4.0	3.8
	Inflammatory arthritis	10.8	10.7	12.9	13.2	13.6	13.9
	Malignancy	4.5	4.6	4.8	4.6	5.9	5.8
	Comorbidity index score^b^	0.5 ± 1.0	0.5 ± 1.0	0.6 ± 1.0	0.6 ± 1.0	0.7 ± 1.2	0.7 ± 1.2
Medications							
	Opioids	55.3	56.1	59.8	59.5	68.2	68.5
	TCAs^c^	10.9	10.4	3.9	3.9	4.8	4.7
	SSRIs	18.4	17.7	22.1	22.6	21.4	21.6
	SNRIs^d^	5.4	5.7	17.8	17.1	8.6	8.7
	Anticonvulsants^e^	12.5	11.8	17.5	17.3	39.0	39.0
	Other antidepressants	11.4	11.7	17.1	17.3	15.4	15.6
	BZDs	29.9	29.7	36.5	36.6	35.2	35.2
	Sleeping disorder drugs^f^	19.9	20.2	23.3	22.6	21.3	21.9
	Migraine drugs	8.3	8.7	7.4	7.7	6.9	6.9
	Muscle relaxants	31.1	31.6	33.2	33.2	39.3	39.6
	Topical analgesics	3.5	3.6	4.5	4.7	6.7	6.8
	Oral steroids	26.5	26.2	26.3	26.3	31.0	31.3
	NSAIDs	33.6	33.4	35.0	34.9	40.4	40.8
	GI protective drugs	21.9	21.9	22.6	22.5	25.2	25.4
Health care utilization^g^							
	Outpatient visits, *n*	7.4 ± 6.9*	7.7 ± 7.3	8.3 ± 7.5	8.2 ± 8.2	8.8 ± 8.4*	8.6 ± 8.1
	PCP visits, *n*	3.6 ± 3.6	3.5 ± 3.5	3.8 ± 3.5	3.7 ± 3.7	3.9 ± 4.2*	3.8 ± 3.7
	Specialist visits, *n*	5.9 ± 7.3*	6.4 ± 8.0	6.5 ± 7.9*	6.8 ± 9.4	7.6 ± 8.8	7.7 ± 9.5
	Prescription drugs, *n*	7.9 ± 5.4	8.0 ± 5.2	8.6 ± 5.4	8.6 ± 5.4	9.4 ± 5.7*	9.2 ± 5.6
	Acute hospitalizations, *n*	9.8*	10.9	9.9*	11.2	14.2*	13.3
	ED visits, *n*	11.8*	10.5	9.9	10.3	12.4*	10.9

^a^BZDs, Benzodiazepines; ED, Emergency department; GI, Gastrointestinal; NSAIDs, Nonsteroidal anti-inflammatory drugs; PCP, Primary care physician; SNRI, Serotonin and norepinephrine reuptake inhibitor; SSRI, Selective serotonin reuptake inhibitor; TCA, Tricyclic antidepressant. Data are expressed as percentage or mean ± SD. ^b^Deyo-adapted Charlson Comorbidity Index [17,18]. ^c^All TCAs except amitriptyline. ^d^All SNRIs except duloxetine. ^e^All anticonvulsants except gabapentin and pregabalin. ^f^Nonbenzodiazepine drugs only. ^g^Not included in the PS model. *Statistically significant difference compared to the reference group (*P* < 0.05).

On average, patients had 7.4 to 8.8 outpatient visits and 5.9 to 7.7 specialist visits during the 180-day baseline period. The mean number of prescription drugs at baseline was also high, between 7.9 and 8.4. Approximately 10% to 12% of patients had at least one ED visit and 10% to 14% had at least one acute hospitalizations during the 180-day baseline period.

### Continuation of fibromyalgia medication

The mean (standard deviation (SD)) duration of treatment was 77 (92) days for the amitriptyline-pregabalin pairs, 94 (110) days for the duloxetine-pregabalin pairs, and 82 (97) days for the gabapentin-pregabalin pairs. The mean (SD) PDCs up to 180 days of follow-up for the amitriptyline and pregabalin pair were 38.6% (30.1) and 50.0% (31.2), respectively. Among the amitriptyline and pregabalin initiators, 7.9% remained on the treatment for at least 180 days. For the duloxetine and pregabalin pair, the mean (SD) PDC values were 55.0% (34.0) and 67.8% (33.4), respectively. Among duloxetine and pregabalin initiators, 16.5% and 8.3%, respectively, remained on the treatment for at least 180 days. The mean PDCs (SD) up to 180 days of follow-up were 45.0% (32.5) for gabapentin initiators and 55.4% (32.4) for pregabalin. Among gabapentin and pregabalin initiators, 9.0% and 9.9%, respectively, remained on the treatment for at least 180 days (Table 2). Of the patients who remained on the treatment for 180 days, the dose was increased in only 5.7% of amitriptyline, 8.7% of duloxetine and gabapentin, and 13.9% of pregabalin initiators.

**Table 2:** **Patients’ continuation of fibromyalgia treatment** ^**a**^

		**Amitriptyline**	**Pregabalin**	**Duloxetine**	**Pregabalin**	**Gabapentin**	**Pregabalin**
Number of patients		8,269	8,269	9,941	9,941	18,613	18,613
Treatment duration							
	0 to 90 days	80.9	79.7	63.5	78.6	78.1	75.7
	91 to 180 days	11.2	12.4	20.0	13.0	13.0	14.3
	181 to 270 days	3.4	4.1	7.6	4.2	4.2	4.9
	271 to 365 days	2.1	1.7	4.0	1.7	2.1	2.2
	Over 365 days	2.4	1.2	4.9	2.5	2.7	2.8
PDC up to 180 days		38.6 ± 30.1	50.0 ± 31.2	55.0 ± 34.0	67.8 ± 33.4	45.0 ± 32.5	55.4 ± 32.4

^a^PDC, Proportion of days covered. Data are percentage or mean ± SD.

### Changes in health care utilization rates

The mean number of outpatient visits and the proportion of patients with at least one hospitalization numerically decreased slightly after treatment initiation compared to before treatment initiation. The number of prescription drugs did not change, and the proportion of patients with at least one ED visit or at least one physical therapy visit increased after treatment initiation in all groups. In the amitriptyline and pregabalin pair, 4.2% of patients taking amitriptyline and 4.5% of patients taking pregabalin were started on another study drug during follow-up. Similarly, 5.6% of duloxetine and 7.1% of pregabalin initiators were prescribed another study drug during follow-up. In the gabapentin-pregabalin pair, 11.5% of gabapentin initiators and 13.8% of pregabalin initiators received another study drug during follow-up. Table 3 shows patients’ unadjusted health care utilization patterns before and after treatment initiation.

**Table 3:** **Crude changes in health care utilization pre- and post–index date among propensity score–matched cohorts** ^**a**^

	**Amitriptyline**		**Pregabalin**		**Duloxetine**		**Pregabalin**		**Gabapentin**		**Pregabalin**	
	**Pre**	**Post**	**Pre**	**Post**	**Pre**	**Post**	**Pre**	**Post**	**Pre**	**Post**	**Pre**	**Post**
Mean number of outpatient visits	7.4	5.5	7.7	6.2	8.3	6.3	8.2	6.5	8.8	6.9	8.6	7.0
Mean number of PCP visits	3.6	2.8	3.5	2.8	3.8	2.8	3.7	2.9	3.9	3.1	3.8	3.1
Mean number of specialist visits	5.9	4.3	6.4	5.0	6.5	4.7	6.8	5.2	7.6	5.8	7.7	5.9
Mean number of prescription drugs	7.9	7.9	8.0	8.3	8.6	8.4	8.6	8.9	9.4	9.3	9.2	9.5
Any acute hospitalization (%)	9.8	5.6	10.9	7.9	9.9	7.7	11.2	8.4	14.2	9.7	13.3	10.5
Any ED visit (%)	11.8	12.4	10.5	13.4	9.9	15.5	10.3	14.5	12.4	17.0	10.9	16.9
Physical therapy (%)	4.4	7.4	5.1	9.7	5.0	7.0	5.2	9.1	6.2	10.8	5.8	10.2

^a^ED, Emergency department; PCP, Primary care physician.

In multivariable Poisson and Cox regression analyses (Table 4), duloxetine initiation was associated with significantly decreased outpatient visits (RR, 0.94; 95% CI, 0.88 to 1.00), prescription drug use (RR, 0.94; 95% CI, 0.90 to 0.86), hospitalizations (RR, 0.75; 95% CI, 0.68 to 0.83), ED visits (RR, 0.85; 95% CI, 0.79 to 0.91), and physical therapy visits (RR, 0.81; 95% CI, 0.71 to 0.92), compared to pregabalin. Amitriptyline initiators had similar rates of health care utilization, except for the significantly lower rate of hospitalization (RR, 0.70; 95% CI, 0.62 to 0.80) and outpatient visits (RR, 0.91; 95% CI, 0.84 to 0.99), compared to pregabalin. Little difference in the rate of health care utilization existed between gabapentin and pregabalin initiators.

**Table 4:** **Adjusted effect of amitriptyline, duloxetine, and gabapentin, compared to pregabalin, on health care utilization during the follow-up period** ^**a**^

**Health care utilization**	**Amitriptyline, RR (95% CI)** ^**b**^	**Duloxetine, RR (95% CI)** ^**b**^	**Gabapentin, RR (95% CI)** ^**b**^
Outpatient visits^c^	0.91 (0.84 to 0.99)*	0.94 (0.88 to 1.00)*	0.94 (0.88 to 1.00)*
PCP visits^c^	0.99 (0.90 to 1.09)	0.92 (0.85 to 0.98)*	0.92 (0.85 to 0.98)*
Any specialist visits^c^	0.85 (0.77 to 0.95)*	0.89 (0.82 to 0.97)*	0.89 (0.82 to 0.97)*
Prescription drugs^c^	0.97 (0.91 to 1.03)	0.94 (0.90 to 0.98)*	0.94 (0.90 to 0.98)*
Acute hospitalization^d^	0.70 (0.62 to 0.80)*	0.75 (0.68 to 0.83)*	0.95 (0.89 to 1.01)
ED visits^c^	0.94 (0.86 to 1.03)	0.85 (0.79 to 0.91)*	1.05 (1.00 to 1.10)
Physical therapy^d^	0.92 (0.80 to 1.06)	0.81 (0.71 to 0.92)*	1.12 (1.03 to 1.21)

^a^CI, Confidence interval; ED, Emergency department; PCP, Primary care physician; RR, Rate ratio. ^b^Pregabalin was used as the reference group. ^c^Multivariable Poisson regression was used. ^d^Multivariable Cox proportional hazards regression was used. *Statistically significant difference (*P* < 0.05). All rate ratios were adjusted for neuropathic pain and the number of outpatient visits, PCP visits, any specialist visits, prescription drugs, acute hospitalizations, and ED visits at baseline.

In a subgroup of patients who received at least 180 days of treatment, amitriptyline, duloxetine, and gabapentin initiators had lower rates of outpatient visits and specialist visits compared to pregabalin initiators (Table 5).

**Table 5:** **Adjusted effect of amitriptyline, duloxetine, and gabapentin, compared to pregabalin, on health care utilization in patients with treatment duration of at least 180 days** ^**a**^

**Health care utilization**	**Amitriptyline, RR (95% CI)** ^**b**^	**Duloxetine, RR (95% CI)** ^**b**^	**Gabapentin, RR (95% CI)** ^**b**^
Outpatient visits^c^	0.91 (0.84 to 0.99)*	0.94 (0.88 to 1.00)*	0.94 (0.88 to 1.00)*
PCP visits^c^	0.99 (0.90 to 1.09)	0.92 (0.85 to 0.98)*	0.92 (0.85 to 0.98)*
Any specialist visits^c^	0.85 (0.77 to 0.95)*	0.89 (0.82 to 0.97)*	0.89 (0.82 to 0.97)*
Prescription drugs^c^	0.97 (0.91 to 1.03)	0.94 (0.90 to 0.98)*	0.94 (0.90 to 0.98)*
Acute hospitalization^d^	0.63 (0.41 to 0.95)*	0.76 (0.57 to 1.01)	0.73 (0.59 to 0.90)*
ED visits^c^	1.07 (0.84 to 1.37)	1.00 (0.83 to 1.19)	1.03 (0.90 to 1.17)
Physical therapy^d^	0.76 (0.55 to 1.06)	0.85 (0.67 to 1.09)	0.99 (0.82 to 1.19)

^a^CI, Confidence interval; ED, Emergency department; PCP, Primary care physician; RR, Rate ratio. ^b^Pregabalin was used as the reference group. ^c^Multivariable Poisson regression was used. ^d^Multivariable Cox proportional hazards regression was used. *Statistically significant difference (*P* < 0.05). All rate ratios were adjusted for neuropathic pain and the number of outpatient visits, PCP visits, any specialist visits, prescription drugs, acute hospitalizations, and ED visits at baseline.

## Discussion

In this large, US population-based cohort study, patients newly prescribed amitriptyline, duloxetine, gabapentin, or pregabalin for fibromyalgia generally had high health care utilization before and after treatment initiation. None of the four fibromyalgia treatments seemed to have much impact on health care utilization; however, duloxetine initiators had significantly fewer outpatient and ED visits and hospitalizations, less prescription drug use, and fewer physical therapy appointments compared to pregabalin initiators. As reported in prior research, depression is a predictor of high health care use [22]. The beneficial effect of duloxetine versus pregabalin seen in this study may be attributable to the fact that duloxetine is also an antidepressant and thus manages depressive mood in patients with fibromyalgia. Amitriptyline initiators also had fewer hospitalizations and physical therapy visits during follow-up than pregabalin initiators did.

Our study highlights several important issues in the management of fibromyalgia. First, fibromyalgia drugs appear to have little effect on reducing health care utilization by patients with fibromyalgia, as seen in previous studies of pregabalin, duloxetine, and TCAs [10-13]. Whether the lack of effect on health care utilization is due to inadequate benefits or concurrent benefit with new side effects of treatment is unclear. It is also difficult to determine whether the use of health care was inappropriately high or clinically necessary. Second, discontinuation rates are high across all study drugs. In previous studies, researchers have found that the effectiveness of these medications is limited [23], and many patients discontinue these medications due to side effects [24]. Third, pharmacologic treatment alone may not be effective in fibromyalgia [25]. Prior meta-analyses of various nonpharmacologic treatments such as massage therapy, aquatic physical therapy, balneotherapy, and hydrotherapy showed mixed results on fibromyalgia symptoms [25-28], although beneficial effects of aerobic exercise and cognitive behavioral therapy on pain and mood were consistently noted [29,30]. Nonetheless, in future longitudinal study, investigators should explore the utility of multifaceted approaches to the treatment of fibromyalgia, including patient education, exercise, and mental health services, with the goal of reducing health care utilization.

There are limitations to our study. First, selection of a drug was not random in this observational cohort. We used rigorous pharmacoepidemiologic methods in the study design and data analysis, such as the new user design and PS-matched analysis, to minimize confounding by indication [19,31]. However, confounding by unmeasured variables, such as severity or activity of fibromyalgia, functional status, other comorbidities, use of nonpharmacologic treatments, and socioeconomic status, can still be an issue. For example, differences existed in comorbidities prior to PS matching, such that duloxetine initiators had a higher proportion of diagnosis and treatment for depression (24%) and anxiety (21%) than the pregabalin group (12% and 13%, respectively) [15]. If the presence or the severity of such conditions, which are related to use of health care systems, were incompletely controlled for, this could lead to residual confounding in our analysis. Second, there was also potential for misclassification, as we relied on diagnosis codes and drug-dispensing records to select patients with fibromyalgia and identify their comorbidities. Third, in this study, the reasons for use of health care resources (for example, outpatient and ED visits, hospitalization) were not available. Fourth, some of the visits to physicians or EDs may not have been related to fibromyalgia. Furthermore, the number of physician visits may not be an appropriate indicator of the effectiveness of fibromyalgia treatment. Fifth, we did not directly compare health care utilization between amitriptyline, duloxetine, and gabapentin initiators. Sixth, although it is possible that pharmacologic treatment has a beneficial effect on health care utilization in patients with fibromyalgia compared to no treatment, our study was designed to examine the comparative effectiveness of the four study drugs. Seventh, because access to health care is different in each health insurance plan in the United States, our results may not be generalizable to populations covered by different health insurance plans, particularly in different countries. Eighth, we did not examine the effect of combination therapy, but less than 10% of patients were put on combination therapy during the study period [15].

## Conclusions

Fibromyalgia patients had substantial health care utilization before and after initiation of amitriptyline, duloxetine, gabapentin, or pregabalin and generally had high discontinuation rates of these medications. Fibromyalgia treatment did not substantially decrease health care utilization. Compared to pregabalin initiators, duloxetine initiators had less health care utilization during follow-up. In future studies, researchers should examine the effectiveness of pharmacologic treatments combined with nonpharmacologic interventions in treating chronic pain, as well as the use of health care resources in fibromyalgia.

## Additional files

Additional file 1:
**Fibromyalgia related drugs.** List of fibromyalgia-related drug names. Additional file 2:
**Algorithms to define comorbidities.** List of diagnosis and procedure codes to define comorbidities.

## Abbreviations

BZD: Benzodiazepine
CI: Confidence interval
ED: Emergency department
FDA: US Food and Drug Administration
GI: Gastrointestinal
ICD: International Classification of Diseases, Ninth Revision, Clinical Modification
NSAID: Nonsteroidal anti-inflammatory drug
PCP: Primary care physician
PDC: Proportion of days covered
PS: Propensity score
RR: Rate ratio
SD: Standard deviation
SNRI: Serotonin and noradrenaline reuptake inhibitor
SSRI: Selective serotonin reuptake inhibitor
TCA: Tricyclic antidepressant
