# Supplementary material for: Patterns of health care utilization related to initiation of amitriptyline, duloxetine, gabapentin, or pregabalin in fibromyalgia
Source: Arthritis Res Ther. 2015 Jan 28;17(1):18. doi: 10.1186/s13075-015-0530-8 (PMC4343277; doi:10.1186/s13075-015-0530-8)
Supplement: Additional file 1: — Fibromyalgia related drugs. List of fibromyalgia-related drug names. [file 13075_2015_530_MOESM1_ESM.docx]

**Additional File 1. Fibromyalgia related drugs**

| **Category** | **Generic drug names** |
| --- | --- |
| Opioids | alfentanil, buprenorphine, fentanyl, hydromorphone, levorphanol tartrate, meperidine, methadone, morphine sulfate, oxycodone, oxymorphone, remafentanil, tramadol, butorphanol, codeine, dihydrocodeine, hydrocodone, nalbuphine, pentazocine, propoxyphene |
|  |  |
| Benzodiazepines | alprozolam, chlordiazepoxide, clonazepam, clorazepate, diazepam, estazolam, flurazepam, halazepam, lorazepam, midazolam, oxazepam, quazepam, temazepam,trizolam, quazepam, prazepam |
| Anti-convulsants | carbamazepine, divalproex, felbamate, gabapentin, lamotrigine, levetiracetam, mephenytoin, phenobarbital, phenytoin, pregabalin, tiagabine, topiramate, valproic acid, vigabatrin, zonisamide |
| TCAs | amitriptyline, amoxapine, clomipramine, desipramine, doxepin, imipramine, nortriptyline, protriptyline, trimipramine |
| SSRIs | paroxetine, sertraline, venlafaxine, fluoxetine, citalopram, nefazodone, mirtazapine, fluvoxamine |
| SNRIs | duloxetine, desvenlafaxine, nefazodone, venlafaxine |
| NSAIDs | celecoxib, diclofenac, etodolac, fenoprofen, flurbiprofen, ibuprofen, indomethacin, ketoprofen, ketorolac tromethamine, mefenamic acid, meloxicam, nabumetone, naproxen, oxaprozin, piroxicam, salsalate sulindac, tolmetin sodium |
| Sleep disorder drugs (non-benzodiazepine) | amobarbital, aprobarbital, barbital/hyoscy/passifl/valerian, butabarb/phenobarb/secobarb, butabarbital, chloral hydrate, eszopiclone, glutethimide, mephobarbital, metharbital, paraldehyde, pentobarbital, phenobarbital, pyrilamine/pentobarbital, ramelteon, secobarbital, zaleplon, zolpidem |
| Other antidepressants | bupropion, isocarboxazid, maprotiline, mirtazapine, phenelzine, selegiline, tranylcypromine, trazodone |
| Topical analgesics | lidocaine patch, capsaicin, lidocaine gel |
| Migraine drugs | almotriptan, dihydroergotamine, eletriptan, ergotamine, frovatriptan, methysergide, naratriptan, rizatriptan, sumatriptan, zolmitriptan |
| Muscle relaxants | carisoprodol, cyclobenzaprine, chlorphenesin, chlorzoxazone, dantrolene, metaxalone, methocarbamol, orphenadrine, baclofen, tizanidine |
| Oral steroids | cortisone, hydrocortisone, prednisone, prednisolone, methylprednisolone, triamcinolone, dexamethasone, bethamethasone |
| GI protective drugs: | cimetidine, ranitidine, famotidine, nizatidine, esomeprazole, lansoprazole, omeprazole, pantoprazole, rabeprazole, sucralfate, aluminum hydroxide, misoprostol, bismuth subcitrate, bismuth subsalicylate |

TCA: tricyclic antidepressant, SSRI: selective serotonin re-uptake inhibitor, SNRI: Serotonin–norepinephrine reuptake inhibitor, BZD: benzodiazepines, NSAIDs: non-steroidal anti-inflammatory drugs, GI: gastrointestinal
