# Supplementary material for: Patterns of health care utilization related to initiation of amitriptyline, duloxetine, gabapentin, or pregabalin in fibromyalgia
Source: Arthritis Res Ther. 2015 Jan 28;17(1):18. doi: 10.1186/s13075-015-0530-8 (PMC4343277; doi:10.1186/s13075-015-0530-8)
Supplement: Additional file 2: — Algorithms to define comorbidities. List of diagnosis and procedure codes to define comorbidities. [file 13075_2015_530_MOESM2_ESM.docx]

**Additional File 2. Algorithms to define comorbidities**

| ***Comorbid conditions*** | ***ICD-9 codes/Procedure codes*** |
| --- | --- |
| Back pain | 720.xx-724.xx |
| Headache | 339.xx, 307.81, 346.x, 784.0x |
| Depression | 296.2x, 296.3x, 311.xx |
| Anxiety | 300.0x, 293.84, 300.2x, 786.01x |
| Seizure | 345.xx |
| Abdominal pain | 564.1x, 789.0x |
| Neuropathic pain | 053.1, 250.6, 337.x,344.6, 650.x,353.x,354.x, 355.x, 357.x, 721.x, 722.x, 729.2 |
| Fatigue | 780.7x |
| Sleep disorder | 327.xx, v69.4x, 780.5x |
| Diabetes mellitus | 250.xx, v45.85 or anti-diabetic medication |
| Hypertension | 401.xx-405.xx. |
| Cardiovascular disease | 410.xx-414.xx |
| Stroke | 430.xx-438.xx |
| Inflammatory arthritis | 714.xx, 720.xx, 696.0, 710.xx |
| Malignancy | 410.xx-209.xx |
| Chronic kidney disease | 580.xx-589.xx, v45.1x |
| Obesity | 278.0x, 278.0x, 649.1, 649.2, 539.x, v45.86, v53.51, 44.38, 44.39, 44.95, 43.89, 44.68, 44.69, 45.51, 45.91, v85.3x, v85.4x  Or use of obesity drugs: sibutramine, phentermine, orlistat, diethylpropion, benzphetamine, phendimetrazine  Or CPT code: 43999, 43770, 43644, 43645, 43845, 43846, 43847, 43842 |
| Smoking | 305.1, 649.0x, 989.84  Or use of drugs: varenicline, bupropion, nicotine  Or CPT code: 99406, 99407, S9075, S9453 |

ICD-9: International Classification of Diseases, 9th revision, CPT: Current Procedural Terminology
